# Polarizability of the active site of cytochrome *c* reduces the activation barrier for electron transfer

**DOI:** 10.1038/srep28152

**Published:** 2016-06-16

**Authors:** Mohammadhasan Dinpajooh, Daniel R. Martin, Dmitry V. Matyushov

**Affiliations:** 1 Department of Physics and School of Molecular Sciences, Arizona State University, PO Box 871504, Tempe, AZ 85287-1504, USA.

## Abstract

Enzymes in biology’s energy chains operate with low energy input distributed through multiple electron transfer steps between protein active sites. The general challenge of biological design is how to lower the activation barrier without sacrificing a large negative reaction free energy. We show that this goal is achieved through a large polarizability of the active site. It is polarized by allowing a large number of excited states, which are populated quantum mechanically by electrostatic fluctuations of the protein and hydration water shells. This perspective is achieved by extensive mixed quantum mechanical/molecular dynamics simulations of the half reaction of reduction of cytochrome *c*. The barrier for electron transfer is consistently lowered by increasing the number of excited states included in the Hamiltonian of the active site diagonalized along the classical trajectory. We suggest that molecular polarizability, in addition to much studied electrostatics of permanent charges, is a key parameter to consider in order to understand how enzymes work.

Cytochrome *c* is an essential redox protein in bacterial photosynthesis and respiratory energy chains of mitochondria. Its redox function is to shuttle electrons between membrane-bound energy complexes, such as between the *bc*_1_ complex and cytochrome *c* oxidase in respiration^1^. The redox activity occurs in heme *c* covalently bound to the polypeptide^2^ (Fig. 1A). The mechanism of transferring the electron, which alters the redox state of the heme, is generally understood within the Marcus theory of electron transfer^3^. It stipulates that the reaction is activated by nuclear fluctuations of the thermal bath, which in the case of protein electron transfer is a highly heterogeneous protein-water interface. The prevailing modes, frequencies, and coupling strengths of those medium modes to the heme’s electronic states are the parameters establishing the overall activation barrier of the reaction^4^,^5^,^6^.

Hydration does not significantly affect vibrational cooling of the heme^7^ and THz absorption of well-hydrated samples is nearly insensitive to the oxidation state^8^. These observations suggest little direct contact of water with the heme^7^. Therefore, cytochrome *c* is a good model system to which basic assumptions of the Marcus theory apply^9^,^10^,^11^. Potential complications of water penetrating the active site^12^ and of conformational transitions upon changing the redox state^2^ are largely insignificant. Our present simulations support this general assessment when applied to the active site represented by fixed atomic charges. However, the main result of this study is the finding that polarizability of the active site extends the problem of protein electron transfer beyond the standard model^13^ by dramatic reduction of the activation barrier.

The established paradigm of the Marcus theory is based on the two-state description involving electronic energy levels of either the electron donor or the acceptor^3^. Nuclear fluctuations of the medium bring these two energy levels into resonance, allowing tunneling to occur^14^. The free energy (reversible work) required to create the resonance condition is determined by two parameters, the reaction free energy Δ*G*_0_ and the medium reorganization energy *λ*. The formulation further simplifies when Δ*G*_0_ is zero for either self-exchange electron transfer or for a half redox reaction occurring at the electrode. The activation free energy (activation barrier) is then fully determined by the reorganization energy^3^


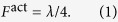


Early calculations and numerical simulations of protein electron transfer produced values of the reorganization energy in the range of 0.7–0.8 eV (see ref. 5 for a review). More recent molecular dynamics (MD) simulations employing improved force fields and significantly longer trajectories resulted in an upward revision of these values toward those more traditional for redox chemistry, ~1.0–1.5 eV^5^,^11^,^15^,^16^ (or even higher^17^). The upward revision of the reorganization energy implies a higher activation barrier in equation (1) and a much slower rate. On the other hand, electrode kinetics measurements typically report much lower values, *λ*~0.4–0.6 eV^18^,^19^, when estimated from the Marcus relation (1). It implies that either the results of numerical simulations for *λ* are grossly incorrect or the relation between the activation barrier and the reorganization energy needs a revision. Here we present arguments that the latter is the case. The revision of the barrier height arises from introducing the polarizability of protein’s active site (Fig. 1B).

Equation (1) can be derived by considering two crossing parabolas *F*_*i*_(*X*) = (*X* ± *X*_0_)^2^/(4*λ*) (*i* = Red, Ox) plotted against the energy gap reaction coordinate *X* as defined by Warshel^20^ (Fig. 1B). The crossing point *F*_1_(*X*) = *F*_2_(*X*) is the transition state of zero energy gap *X* = 0, where tunneling occurs^21^,^22^,^23^. The Marcus formulation follows from requiring *X*_0_ = *λ* as stipulated by the fluctuation-dissipation theorem^24^.

Recent simulations have shown that proteins are often unable to sample their entire phase space on the reaction time-scale. This ergodicity breaking^6^ eliminates the restriction on the reaction parameters imposed by the fluctuation-dissipation theorem^25^. In particular, *X*_0_ and *λ* become two separate parameters^6^,^26^ and finding the activation barrier requires three parameters (*X*_0_, *λ*, and Δ*G*_0_), instead of two parameters of the Marcus theory. Sampling of the entire phase space is never realistically possible, but the problem is drastically elevated for proteins because of their rugged energy landscape, similar to those found for fragile glass formers^27^,^28^. The dynamics and statistics of proteins are characterized by many local minima, in which the protein-water system can be trapped, never reaching its true thermodynamic minimum^29^. Electron transfer reactions between non-equilibrium trapped states do not follow the strict restriction *X*_0_ = *λ*^6^.

The direct mechanistic consequence of this new perspective is an extended flexibility in fine-tuning the activation barrier of electron transfer^6^,^30^. Since *X*_0_ refers to the average of the vertical transition energy, it defines the position of the maximum of an optical spectroscopic line^31^ and can be associated with the Stokes shift of optical spectroscopy. One can therefore define the Stokes shift reorganization energy as *λ*^St^ = *X*_0_^13^. The three-parameter description leads to the following activation barrier when Δ*G*_0_ = 0^6^


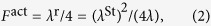


where the “reaction reorganization energy” *λ*^*r*^ can be identified with the Marcus reorganization energy in equation (1). *λ*^*r*^ = (*λ*^St^)^2^/*λ* is in fact consistent with the standard Marcus definition of the reorganization energy^3^ as the free energy invested to shift the system to the position of the products (thus moving 2*λ*^St^ along *X*) while remaining on the free energy surface of the reactants characterized by the curvature (2*λ*)^−1^.

The reorganization energy in the denominator in equation (2) is defined as the variance of the reaction coordinate


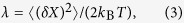


where *k*_B_ is the Boltzmann constant and *T* is the temperature. Note that long trajectories, >100 ns or longer^32^, are required to converge *λ* (see Supplementary Fig. S6). Because of this difficulty, most simulations, with few exceptions^5^, report *λ*^St^ instead of *λ*.

It is clear that the activation barrier can be lowered compared to equation (1) of the Marcus model when *λ*^St^ < *λ*. The parameter


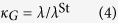


quantifies the difference between two reorganization energies in the three-parameter model^26^. Note that electrochemical kinetic measurements report *λ*^r^. The low values of such effective reorganization energies^18^,^33^,^34^ are therefore consistent with *κ*_*G*_ > 1 as schematically shown in Fig. 1B. It is also clear that the rate maximum, when the rate is plotted against the driving force −Δ*G*_0_ (the Marcus inverted parabola^3^), gives the value of *λ*^St^ = *X*_0_ only and provides no access to *λ*.

Important for biological applications is that *κ*_*G*_ > 1 lowers the activation barrier without requiring more negative reaction free energy, which is a scarce commodity in biological energy chains^1^. It seems therefore possible that the evolutionary pressure has favored the glassy character of the protein fluctuations, and their high fragility^27^, to promote electron transport consuming less free energy input for its operation.

The Marcus formulation of the electron-transfer theory can be viewed as the first-order quantum-mechanical perturbation of the electronic energy levels by the thermal bath. The perturbation Hamiltonian comes from integrating the electronic density *ρ*_*e*_(**r**) with the electrostatic potential of the bath *ϕ*(**r**): 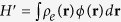. When the electronic density is given by a set of atomic charges *q*_*α*_, one arrives at the force-field formulation often implemented in classical simulations. The solute-solvent Hamiltonian is obtained by summing up partial atomic charges with the bath potentials at their locations: 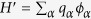 (Fig. 1C). However, fluctuations of the medium not only alter the donor-acceptor energy gap (between HOMO and LUMO), but also the entire manyfold of the electronic energy states. Each instantaneous nuclear configuration of the medium will produce a different extent of electronic delocalization between those available electronic states, or, alternatively, a different deformation of the electronic density.

The ability of the electronic distribution to deform in an external field is associated with its electronic polarizability. In the dipolar approximation, it is given in terms of transition dipoles *μ*_*km*_ linking different electronic sates of the molecule through the electric field of the bath *E*_*b*_ (Fig. 1C). The standard quantum-mechanical perturbation theory leads to the quadratic Stark effect^35^, shifting the energy level *k* by the amount 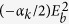 scaled with the polarizability of that state


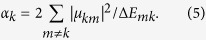


It is determined by a set of transition dipoles and energy gaps Δ*E*_*mk*_ = *E*_*m*_ − *E*_*k*_ of all possible virtual excitations.

Perturbation theory is not required to introduce polarizability into the description of electron transfer. A more accurate formalism is achieved by using the empirical valence-bond approximation introduced by Warshel and Weiss^36^,^37^,^38^. It produces the instantaneous energies of the donor and acceptor by diagonalizing the Hamiltonian matrix incorporating the coupling to the medium into the diagonal (electrostatics) and off-diagonal (transition dipoles) matrix elements. This approach has been widely used for a number of biologically relevant systems in the past^39^,^40^ and has recently been implemented in the form of the perturbed matrix algorithm^11^ in application to protein electron transfer. We follow this general formalism in the simulations presented in this paper. Our main goal is to explore the possibility of lowering the barrier for electron transfer by including mixing between the quantum states (polarizability). From the more fundamental perspective, our study asks the question of whether including polarizability of the enzyme’s active site, in contrast to the picture of fixed atomic charges, might reduce the barrier of an enzymetic reaction. In other words, the question is whether polarizability is one of the “tools” of biology’s catalytic capability^41^.

## Results

### QM/MD simulations

**Figure 1:**
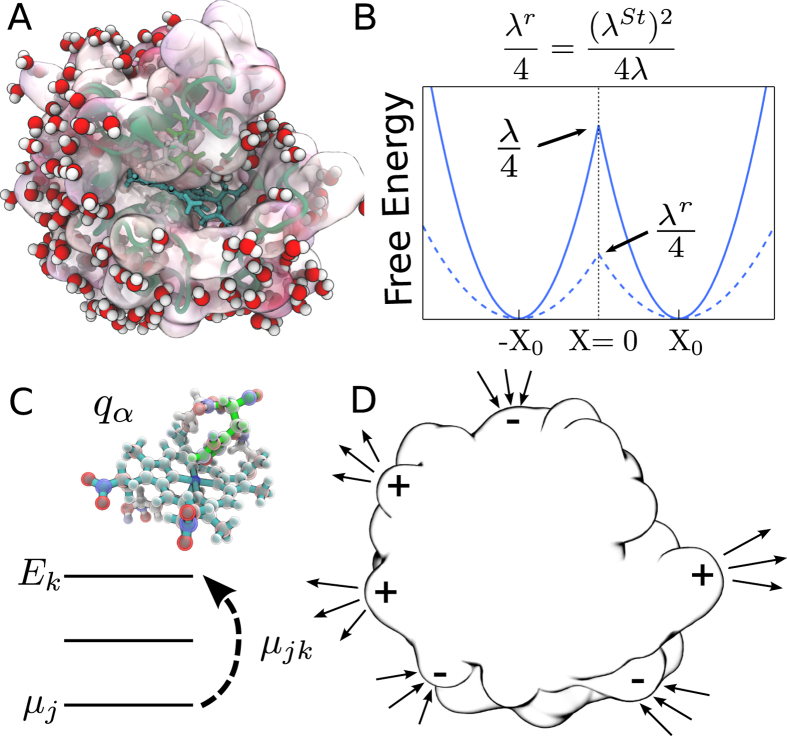
(**A**) Hydrated cytochrome *c* with the heme active site shown in green. (**B**) The free energy surfaces of a half reaction in the Marcus model (equation (1)) of fixed atomic charges (solid lines) and in the polarizable model with *λ*^St^ < *λ* (equation (2)). (**C**) The active site representation by atomic charges *q*_*α*_ in classical simulations and by a Hamiltonian matrix coupled to the classical bath in QM/MD simulations. (**D**) The mechanism of compensation of protein and water electrostatics through polarizing the interfacial water dipoles by the charged residues of the protein.

The goal of our simulation strategy is to go beyond the assumption of fixed atomic charges in the modeling of the redox active site. We introduce the ability of the electronic density of the heme in cytochrome *c* to redistribute in response to a thermal fluctuation of the bath. This goal is shared by essentially all QM/MM algorithms which all start by defining the quantum center, i.e., a part of the system which can be treated on the quantum-mechanical (QM) level^5^,^30^,^40^,^42^,^43^,^44^. The choice of the level of QM calculations is dictated by the physics of the problem and, to a large degree, by the time-scale required to capture the essential collective modes of the thermal bath contributing to the activation barrier^45^. Protein electron transfer is a difficult problem for QM algorithms because long time scales are very essential here. Classical simulations of electron transfer have shown that a broad range of bath time-scales affects the reorganization energy^5^,^26^. The time-scales of ~1 ns represent global elastic deformations of the protein shape, which have to be included for a realistic description of *λ*. These motions produce large fluctuations of electrostatic potential inside the protein by shifting charged surface residues and surface water polarized by them^6^ (Fig. 1D). As more elastic modes enter the observation window (the length of the simulation trajectory), the reorganization energy grows nearly continuously through the range of time-scales up to tens of microseconds currently reached by simulations^32^. Given these constraints imposed by the physics of the problem, a QM algorithm needs to capture the entire range of thermal motions sampled by classical simulations.

The method of perturbed matrix^11^ imposes essentially no QM overhead on the classical MD. It assumes that the forces acting on the atoms of the classical thermal bath can be well characterized by classical force fields. One therefore starts with a long classical MD simulation of the entire system producing the dynamics of the classical bath. These classical dynamics are then used to recalculate the parameters of the quantum center affected by the electrostatic interactions with the bath. Since long-range electrostatics is the main factor influencing the positions of the donor and acceptor energy levels involved in electron transfer^4^, this algorithm is particularly well suited for this problem.

The choice of the QM center (Fig. 1C and Supplementary Fig. S4) is explained in detail in the Supplementary Information. Briefly, several QM centers of increasing size have been tried. The largest QM center adopted for the analysis includes the heme, histidine, methionine, and two cysteine amino acids. As we show in Supplementary Table S1, there is a fairly insignificant change in the spectrum of electronic states between this QM center and a somewhat smaller one, without two cysteine residues.

The QM component of the analysis was performed by expanding the electrostatic potential of the bath *ϕ*(**r**) around the potential *ϕ*_Fe_ at the heme iron and truncating the expansion at the dipolar term. The matrix elements of the quantum center Hamiltonian then become


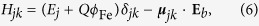


where *Q* is the total charge of the quantum center. The quantum states *j* = 0,…, *M* include the ground state of the quantum center, *j* = 0, and a number of its excited states produced here by ZINDO/S calculations for the oxidized (Ox, *Q* = −1) and reduced (Red, *Q* = −2) states. The polarizability is a slowly converging function of the number of excited states *M*^46^; the results presented here were obtained for *M* = 100. Reducing *M* makes the quantum center less polarizable and eventually brings the system back to the Marcus domain. This was the result of a recent calculation employing *M* = 12^11^.

The Hamiltonian matrix in equation (6) is diagonalized at each instantaneous value of the potential *ϕ*_Fe_ and the electric field **E**_*b*_ along the simulation trajectory to produce the minimum eigenvalues 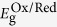 corresponding to the ground state in either oxidized or reduced states of the active site. The electron-transfer reaction coordinate, monitoring the transition to the activation state *X* = 0, is given as^20^


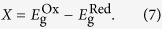


The limit of classical simulations is obtained by representing the quantum center by a set of atomic charges coupled to the bath through the corresponding electrostatic potentials *ϕ*_*α*_ (Fig. 1C). The reaction coordinate of electron transfer becomes in this case


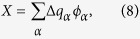


where 
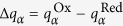
. More details on the protocols of quantum calculations and of classical simulations are provided in Methods below and in the Supplementary Information. Here we discuss the results of our analysis.

### Free energy surfaces of electron transfer

**Figure 2:**
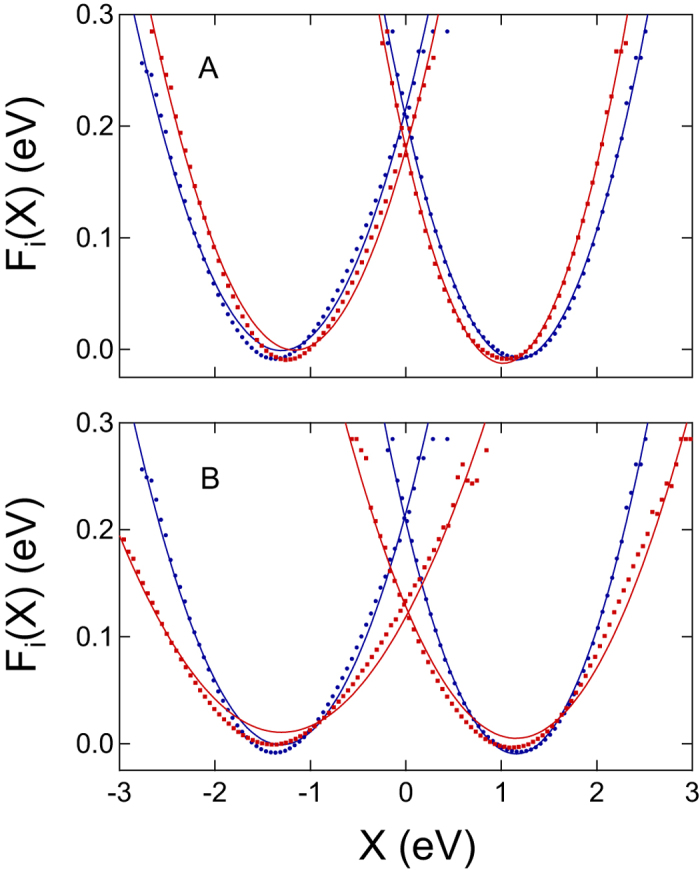
Free energy surfaces *F*_*i*_(*X*), *i* = Red, Ox of cytochrome *c* in the Ox (left curves) and Red (right curves) states. The blue points/lines refer to classical MD and the red points/lines refer to the QM/MD simulations. The solid lines are fits of the simulation data to parabolas. Panel (A) refers to a non-polarizable quantum center (*ξ* = 0). Panel (B) refers to a polarizable quantum center with *ξ* = 1 and Δ*α* = −31 Å^3^. The lower panel demonstrates the depression of the barrier height upon allowing a non-zero *α*_*k*_ (see Supplementary Fig. S7 for *ξ* = 2, Δ*α* = −123 Å^3^).

The free energy surfaces of electron transfer *F*_*i*_(*X*) = −*k*_B_*T* ln[*P*_*i*_(*X*)] (*i* = Ox, Red) follow from probabilities *P*_*i*_(*X*) calculated from classical trajectories with the quantum (equation (7)) or classical (equation (8)) definitions of the reaction coordinate *X*. Since our focus is on a half reaction, we do not consider a more complex problem of calculating the redox potential^5^,^43^ and focus solely on the reorganization energies. We first note that the quantum and classical algorithms are consistent with each other when the polarizability of the active site is turned off (Fig. 2A). In order to study the effect of the active site polarizability, we introduced scaling of the transition dipoles with the scaling factor *ξ*: ***μ***_*mk*_ → *ξ**μ***_*mk*_. The non-polarizable active site corresponds to *ξ* = 0 when coupling between the quantum states is turned off. Even in this limit, the algorithms of calculating *X* are still somewhat different in the quantum and classical cases since we use an expansion of the potential in the quantum Hamiltonian in equation (6), in contrast to a full set of atomic charges in the classical MD. However, the free energy surfaces obtained in these two approaches are consistent (Fig. 2A), suggesting a little effect of re-distributing the charge within the active site on the electron-transfer barrier. One can further examine the effect of charge distribution in the active site by assuming complete localization of the charge on heme’s iron, Δ*q*_Fe_ = 1. This extreme case is compared to the calculation with distributed charge in Table 1. The results are consistent.

Little sensitivity to electron delocalization might seem to be a trivial outcome since standard electrostatics suggests that the interaction of a point charge (localization) with the surrounding medium is equivalent to that of a charge uniformly spread over the conducting surface (delocalization). However, it is often suggested that delocalizing the electronic density of the active site is an optimization mechanism to reduce the reorganization energy^43^. While this mechanism is expected to lower the reorganization energy of localized skeletal vibrations^2^,^15^, we see little evidence for it altering the medium (protein and water) reorganization energy. It is also useful to keep in mind that most reactions relevant to biology’s energy chains occur at relatively small magnitudes of the driving force −Δ*G*_0_ and, therefore, proceed in the normal region of electron transfer when quantum vibrations have essentially no effect on the activation barrier^47^. We instead argue here that the reorganization energy *λ* is maximized, and not minimized, for polarizable active sites. Since *λ*^St^ remains nearly unaffected, the activation barrier in equation (2) can be reduced (Fig. 1B) due to a large value of the parameter *κ*_*G*_ (equation (4)).

### Effect of polarizability on the reorganization energy

The main goal of our analysis is to establish whether *λ* can significantly exceed *λ*^St^ when polarizability of the protein’s active site is turned on. We first note that 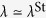, in accord with the standard Marcus theory^3^, in the classical MD simulations (Table 1). This result is in agreement with previous simulations of this protein^9^,^11^, although we still find *κ*_*G*_ > 1. A reasonable agreement with the Marcus theory found here is not always shared by other redox proteins. We have found *λ* > *λ*^St^ for a number of redox proteins (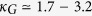 for electron transfer reactions in bacterial reaction centers^6^,^26^,^32^). The reasons why non-polarizable cytochrome *c* falls on the lower end of *κ*_*G*_ values are important to understand. We discuss below the mechanism of compensation between the protein and water fluctuations lowering *κ*_*G*_. Here we first look at how altering the physical model from a set of fixed atomic charges to a fluctuating charge distribution affects the activation barrier.

**Figure 3:**
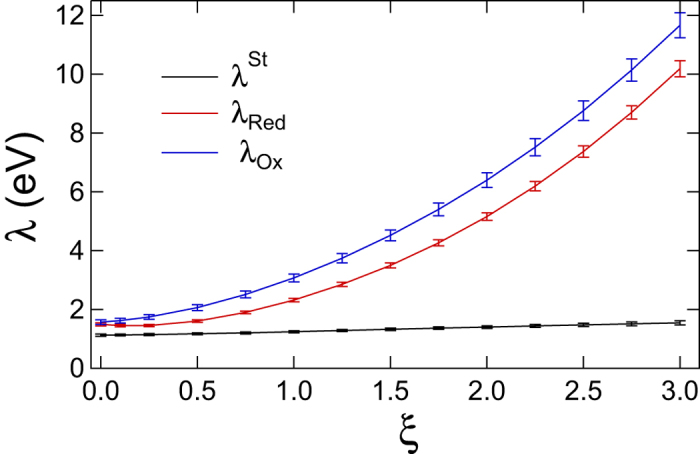
Reorganization energies *λ* and *λ*^St^ against the scaling factor altering the transition dipoles as *μ*_*km*_ → *ξμ*_*km*_. The points are the results of calculations with error bars shown and the lines are regressions through the point to guide the eye.

Table 1 and Fig. 3 summarize our findings. The reorganization energy *λ* is calculated according to equation (3), while 2*λ*^St^ = 〈*X*〉_Red_ − 〈*X*〉_Ox_ is calculated from the average energy gaps in two redox states. The polarizability of the quantum center is continuously increased in Fig. 3 by scaling the ZINDO/S transition dipoles, ***μ***_*jk*_ → *ξ**μ***_*jk*_. The corresponding polarizabilities, calculated from equation (5), are listed in Table 1. As mentioned above, the polarizability significantly drops when fewer states are included and the statistics of the electron-transfer energy gap returns back to 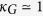 of the Marcus theory (Supplementary Table S2). Even with *M* = 100 excited states, our calculations are likely to underestimate the polarizability of the active site^46^. The scaling parameter *ξ* > 1 allows us to estimate the potential effect of an increased polarizability on the reaction activation barrier.

Increasing the polarizability clearly separates *λ*^Ox/Red^ from *λ*^St^ (Fig. 3). According to equation (2), this should lower the activation energy, as is also seen from direct calculations shown in Fig. 2B. The main result of our calculations is that electron transfer involving polarizable active sites should proceed with lower activation barriers, without requiring more negative reaction free energy. Why this is the case can be understood from the following general arguments. The reversible work of creating a fluctuation of the bath field is a quadratic function of the field, 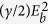, in linear response. The negative free energy invested in polarizing the solute reduces this energy as 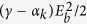. One expects, therefore, a smaller activation barrier to reach *X* = 0, as we observe. There is also a possibility of breaking the harmonic stability at 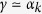, when *λ* passes through a spike^48^. Since *λ* enters the denominator in Eq. (2) and *λ*^St^ is not much affected by polarizability (Fig. 3), one can anticipate that an electron-transfer enzyme can reach its lowest activation barrier if operating in the regime 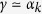.

### Electrostatics of protein and water

The overall reorganization energy is a gauge of the strength of thermal fluctuations affecting the active site (equation (3)), with water and protein being its two main components. It is therefore of great mechanistic interest to understand what are the relative contributions of protein and water to fluctuations experienced by the active site. In contrast to some early suggestions that soluble proteins can effectively screen water from the active site and thus produce an effectively nonpolar environment, a number of recent simulations have clearly shown that water can never be neglected^5^,^49^,^50^ and reorganization energies are comparable in magnitude to those traditionally reported for synthetic donor-acceptor complexes^5^,^11^,^16^,^32^. These new results are in line with the current shift of the view on the relation between proteins and water suggesting that water should be viewed as an integral part of the protein^51^. However, an upward revision of the reorganization energy for protein electron transfer raises an important mechanistic question of how high efficiency of biological energy chains is achieved.

**Figure 4:**
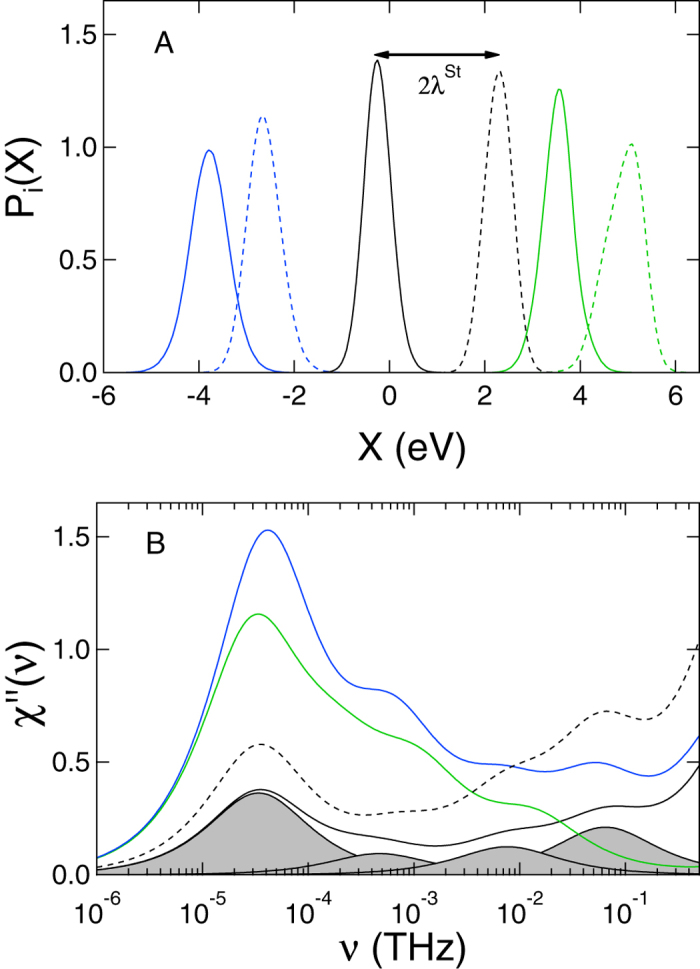
(**A**) Distribution of the electron-transfer coordinate (black) and its protein (green) and water (blue) parts (classical MD). The solid lines refer to the Ox state and the dashed lines refer to the Red state. The distance between the maxima of Red and Ox distributions is the Stokes shift, 2*λ*^St^. (**B**) Loss function *χ*′′(*ν*), 2*πν* = *ω* from protein (green), water (blue), and total (black) fluctuations of *X*. The solid black line shows the classical MD and the dashed line represents the QM/MD simulations at *ξ* = 1 (Table 1). The loss functions are normalized to give the corresponding reorganization component from 
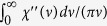
. The shaded areas represent separate Debye relaxation processes used to fit the time correlation function *C*_*X*_(*t*) from classical MD simulations.

One first needs to realize that there is a significant screening between the water and protein contributions to the electron-transfer energy gap. The water dipoles are oriented by the ionized surface residues of the protein to produce the electrostatic potential opposite in sign to the potential of the protein (Fig. 1D). As a result, the protein (p) and water (w) contributions to *X*_0_ = 〈*X*_*w*_〉 + 〈*X*_*p*_〉 are typically opposite in sign and similar in magnitude (Fig. 4A). The value of *X*_0_ is the result of their incomplete compensation. The same physics applies to the variance of *X*, that is to the reorganization energy in equation (3).

The reorganization energy obtained from equation (3) becomes the sum of three components: protein, *λ*_*p*_, water, *λ*_*w*_, and a cross component, *λ*_*pw*_ = 〈*δX*_*p*_*δX*_*w*_〉/(*k*_B_*T*), produced by correlated protein and water fluctuations. Consistent with the opposite signs of 〈*X*_*p*_〉 and 〈*X*_*w*_〉, the cross component is negative and compensates much larger individual protein and water contributions^52^. For instance, for *λ*_Ox_ = 1.67 eV listed in Table 1, one has *λ*_*p*_ = 2.28 eV, *λ*_*w*_ = 3.39 eV and *λ*_*pw*_ = −4.0 eV (Supplementary Table S3).

### Dynamics

The compensation between the protein and water fluctuations, displayed in the overall value of *λ*, shows itself even more dramatically in the Stokes shift dynamics of the energy gap variable *X*(*t*). To study the dynamics, one has to turn to time correlation functions. The simplest one is the binary auto-correlation function *C*_*X*_(*t*) = 〈*δX*(*t*)*δX*(0)〉, where *δX*(*t*) = *X*(*t*) − *X*_0_. The *t* = 0 value of this correlation function is proportional to the reorganization energy and one can anticipate that the physics of protein-water electrostatic compensation should extend into the time domain. It does, but we also find new dynamics pertinent to each component, which loses its prominence in the overall Stokes-shift dynamics due to the compensation effect.

Figure 4B shows the loss spectrum of the Stokes-shift dynamics. The loss function *χ*′′(*ω*) characterizes the rate of energy exchange, at a given frequency, between the active site and the thermal bath. It can be thought of as the rate of energy dissipation, at a given frequency, of some energy (e.g., photon) absorbed by the active site. The overall energy dissipated into surrounding is the integral of *χ*′′(*ω*)/*ω* over all frequencies. In our calculations, 2*k*_B_*Tχ*′′(*ω*) = *ωC*_*X*_(*ω*) is obtained from the frequency Fourier transform of the time correlation function^24^.

The peaks of *χ*′′(*ω*) show the characteristic relaxation times of the modes coupled to the electron-transfer coordinate and their intensities represent the coupling strengths. The main striking observation from the plot is the presence of slow dynamics in both the protein and water components characterized by nearly equal relaxation times. These common dynamics, in the nanosecond time domain (see SI), represent elastic modes altering the shape of the protein and simultaneously shifting the surface water molecules oriented by charged protein residues (Fig. 1D)^6^. The slow dynamics, however, nearly disappear in the overall *χ*′′(*ω*) due to a strong compensation (screening) between protein and water electrostatic contributions. It is this compensation that brings *λ* in a near accord with *λ*^St^ in the case of non-polarizable heme of cytochrome *c*. The lack of this compensation makes the two reorganization energies deviate from each other, often significantly, for other proteins^6^.

### Mechanistic aspects

Our QM/MD calculations produce the effective reorganization energy in equation (2) 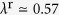 eV, *λ* = (*λ*_Ox_ + *λ*_Red_)/2 not far from ~0.6 eV viewed to be the average number from solution-based measurements^19^. The error bars of the reorganization energy calculations are displayed in Fig. 3 and are additionally listed in Supplementary Table S3. The errors of reorganization energy calculations from QM/MD do not exceed 4%.

One still has to be aware that the present simulations do not directly include polarizability of water and molecular groups of the protein in the production runs. A number of recent simulations employed polarizable force fields^5^,^30^,^45^,^50^. The polarizability is included *a posteriori* in our analysis of the trajectories by calculating the energy of induction interaction of the electric field of the QM center with the induced dipoles of the protein and water^49^,^53^ (see Supplementary Information for details). The induction (bath polarizability) component of *X*_0_ can be significant, but it mostly cancels out^54^ in *λ*^St^. The remaining nonzero contribution to the reorganization energy is caused by density fluctuations modulating the induction interaction energy.

The reorganization energies can potentially decrease if induced dipoles are included. The Pekar factor of dielectric models predicts a drop of *λ* by the factor 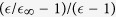 when switching from a nonpolarizable to a polarizable dielectric; 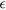 and 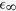 are, correspondingly, the static and high-frequency dielectric constants of the thermal bath. However, simulations of model systems^55^ show that this drop is an overestimate and the reorganization energy decreases only by ~20% upon the inclusion of induced dipoles. All these results apply, however, to the Marcus picture with *λ*^St^ = *λ*. It is currently unclear how induced dipoles affect each distinct reorganization energy *λ*^St^ and *λ*. In addition, a drop in the magnitude of the reorganization energy upon including induced dipoles is mostly off-set by the reorganization energy arising from translational motions of induced dipoles (induction reorganization energy, see Supplementary Information).

As mentioned above, *κ*_*G*_ > 1 requires either incomplete sampling (ergodicity breaking), when some configurations are not accessible, or the breakdown of the Gaussian statistics of the energy gap fluctuations and non-parabolic free energy surfaces. The latter scenario is indeed realized for donor-acceptor systems with polarizabilities different between the two electron-transfer states^13^. However, this scenario requires *λ*_Ox_ ≠ *λ*_Red_. This seems to be generally true for polarizable active sites (Table 1), but the extent of deviation is hard to estimate with limited sampling available from protein simulations. We also note that the dynamics of the energy gap *X*(*t*) follow the Gaussian approximation. It is tested by the ability to produce the fourth-order time correlation function of the energy gap in terms of the Stokes shift dynamics (Supplementary Fig. S8)^56^. Overall, we cannot clearly assign *κ*_*G*_ > 1 found in our simulations to the non-Gaussian character of the energy gap fluctuations.

## Discussion

Energy chains of biology rely on a very short list of redox centers to transfer electrons^1^. They mostly include hemes of cytochromes, iron-sulfur clusters, and cupredoxins. One wonders if they are used to allow distinctly different electron-transfer mechanisms or have been selected based on similar mechanistic properties. A partial answer comes from biology. Cytochrome *c6*, a heme protein, is used interchangeably with plastocyanin, a cupredoxin, in cyanobacteria to catalytically connect photosystems I and II^2^ (only plastocyanin is used in higher plants). Numerical simulations have shown that *λ* ≫ *λ*^St^, attributed in this study to a high polarizability of the active site, is achieved in plastocyanin through insufficient compensation between water and protein electrostatics, which does not require a polarizable active site^26^. Does it mean that evolutionary pressure chooses redox proteins with *λ* ≫ *λ*^St^, regardless of the mechanism producing the desired result? We do not have a definitive answer at this time. Studies of the effect of polarizability on electron transfer in all three classes of redox centers are required to address this question.

What our study convincingly shows is that increasing the polarizability of protein’s active site can significantly reduce the activation barrier of a catalytic reaction, electron transfer in this case. Interaction of atomic charges of the active site with the electrostatic potential of the surrounding medium is clearly an essential part of the enzyme’s catalytic action^57^. It might be true as well that not only the distribution of molecular charge, but also its ability to deform in the external field (polarizability) is an important “tool” employed by nature to catalyze biological reactions.

Polarizability of an active site is controlled by a set of transition dipoles and energy gaps between the state of the transferred electron and the excited electronic states (equation (5)). Minimizing the energy gaps and maximizing the transition dipoles enhances the polarizability. The primary pair of bacterial photosynthesis (a sandwich of closely spaced and parallel oriented bacteriochlorophylls^58^) is a dramatic example showing that polarizability can be effectively manipulated by design. The two bacteriochlorophylls form a partial charge transfer state with a strong electronic coupling and a low energy gap between the eigenstates^49^. The resulting polarizability of the photoexcited state^59^, ~10^3^ Å^3^, is among the highest in the molecular world, and it affects the energetics of primary charge separation^49^. The active site polarizability can be viewed as an additional “knob” turned by evolutionary pressure through chemical manipulation of the excited electronic states.

## Methods

The NMR solution structure of horse heart cytochrome *c* (PDB 1GIW) was adopted as the starting configuration for classical MD simulations. The simulations were done with NAMD software suite^60^, with the trajectory length of 250 ns and 33231 TIP3P water molecules in the simulation box. Additional details of the simulation protocol and of the quantum calculations are given in the Supplementary Information.

## Additional Information

**How to cite this article**: Dinpajooh, M. *et al*. Polarizability of the active site of cytochrome *c* reduces the activation barrier for electron transfer. *Sci. Rep.* **6**, 28152; doi: 10.1038/srep28152 (2016).

## Supplementary Material

Supplementary Information

## Figures and Tables

**Table 1:** Reorganization energies (eV)^a^.

Method	*λ*^St^	*λ*_Ox_	*λ*_Red_	*κ*_*G*_^b^
	Classical			
Classical MD	1.26	1.67	1.64	1.3
Δ*q*_Fe_ = 1^c^	1.13	1.57	1.50	1.4
*α*_Red_, Δ*α*/Å^3^ (*ξ*)^d^	Quantum Mechanical			
0.0, 0.0(0)	1.13	1.57	1.50	1.4
54, −31(1)	1.24	3.07	2.32	2.2
216, −123(2)	1.40	6.40	5.16	4.1
1, −3^e^	0.89	0.92	1.32	1.3

^a^The error bars are ±(0.04–0.06) eV for the classical calculations and ±(0.04–0.2) eV for the quantum calculations. More details can be found in Supplementary Table S3.

^b^*κ*_*G*_ is defined by equation (4).

^c^Calculated from the variance of electrostatic potential at the heme iron thus assuming that charge is fully transferred to the heme iron in the half reaction.

^d^The difference of the quantum center polarizability in the Ox and Red states calculated from equation (5); the number in the bracket is the factor scaling the transition dipole moments, ***μ***_*km*_ → *ξ**μ***_*km*_ (also see Fig. 3).

^e^The results of simulations from ref. 11, Δ*α* is estimated from the present calculations based on *M* = 10.
