# Supplementary material for: Polarizability of the active site of cytochrome c reduces the activation barrier for electron transfer
Source: Sci Rep. 2016 Jun 16;6:28152. doi: 10.1038/srep28152 (PMC4910110; doi:10.1038/srep28152)
Supplement: Supplementary Information [file srep28152-s1.pdf]

Polarizability of the active site of cytochrome c reduces the activation barrier for electron transfer. Supporting Informaton

Mohammadhasan Dinpajooh, Daniel R. Martin, and Dmitry V. Matyushov

Department of Physics and School of Molecular Sciences, Arizona State University, PO Box 871504, Tempe, AZ 85287-1504 [a)]

Classical Molecular Dynamics (MD) Simulations. The NMR solution structure of reduced horse heart cytochrome c (PDB 1GIW) was adopted as the starting configuration for classical MD simulations. The CHARMM 27[1] force field was used for the peptide chain, while the parameters for the heme group in the reduced (Red) and oxidized (Ox) states were adopted by combining atomic charges from Leu et al[2] with the bonded and van der Waals parameters from Kaszuba et al[3]. Patches were applied to connect the heme group to the protein matrix through ligation of two cysteine residues (res. No 14 and 17) and a single methionine residue (No 80).

Most electron-transfer cytochromes form 6-coordinated His-Fe-Met complex[4]. The Fe-His bond is, however, weaker than the Fe-Met bond and can break in some forms of cytochrome c[5]. The stretching frequency of the Fe-His bond in 6-coordinated cytochromes is $\sim 220-240$ cm^{-1}[5]. The breaking of the Fe–Nϵ bond was previously modeled by QM/MM simulations[5] and the resulting potential is shown by points in Fig. S1. A Morse potential was used to represent the results of the simulations

$$U(r) = D_e \left[1 - e^{-\gamma(r-r_e)}\right]^2 \qquad \text{(S1)}$$

with the well depth $D_e = 9.0$ kcal/mol, the well width $\gamma = 1.52$ Å^{-1}, and the equilibrium bond distance $r_e = 2.33$ Å (Fig. S1). The potential in Eq. (S1) was applied to the simulations in the form of the force by utilizing NAMD tclForces functionality. The bond does not break during the simulation time, but the Morse potential allows additional flexibility of the system along the bond stretch coordinate.

From the original PDB structure, crystallographic water molecules were taken from the 1YCC PDB file and, after aligning the two protein structures, were added to the 1GIW cytochrome c structure. To assure that the protein was properly saturated with water, we performed a "soaking" procedure. It consisted of making a small sphere of water surrounding the protein with a total system size of 5497 atoms. From this structure, 150 ns simulations were performed. Finally, from the last frame of these longer simulations, a box (100.1Å×100.1Å× 100.1Å) consisting of a total of 101440 atoms was created and additional water molecules added to the total of 33231 molecules. This addition of water was followed by 20 ns NPT simulations allowing the newly created box to relax around the sphere. This NPT equilibration was followed by 10 ns NVT equilibration for each redox state. All force field parameters were applied using VMD's "psfgen" tool and TIP3P water molecules were added using VMD's "solvate" plugin[6].

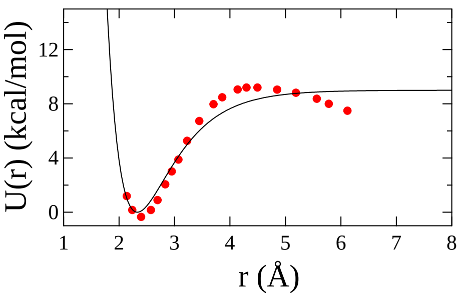

FIG. S1. Morse potential modeling the Fe–Nϵ bond. The red points are from Ref. 5 while the black line represents the Morse potential in Eq. (S1) with $D_e = 9.0$ kcal/mol, $\gamma = 1.52$ Å^{-1}, and the equilibrium bond distance $r_e = 2.33$ Å.

All simulations were performed using NAMD software program[6]. For all initial systems, a steepest descent minimization was performed for 2000 steps. The NPT equilibration simulation was done using the Langevin dynamics in NAMD with the following parameter set: a damping coefficient of 1 ps^{-1}, piston period of 100 fs, the piston decay time of 50 fs, the piston target pressure of 1.01325 bar, and constant temperature control set to 300 K. The NVT simulations were performed using the same parameters as the NPT simulations, but removing the constant pressure controls. Long-range electrostatic interactions were treated with the particle mesh Ewald technique using a cutoff distance of 12.0 Å. A 2.0 fs time step was used for all simulations. 250 ns MD simulations were carried out for trajectories production. Additional 10 ns simulations were performed with the saving frequency of 8 fs to study the short time dynamics. The stability of the protein backbone structure is represented by RMSD values of the backbone α-carbons in Fig. S2A; the RMSDs of the Fe atom in the two oxidation states are shown in Fig. S2B.

Figure S3 shows the iron-oxygen pair distribution function in the Ox and Red states of the protein. One can detect the presence of a water molecule next to the heme in the Red state. This water molecule leaves the heme

[a)] Electronic mail: dmitrym@asu.edu

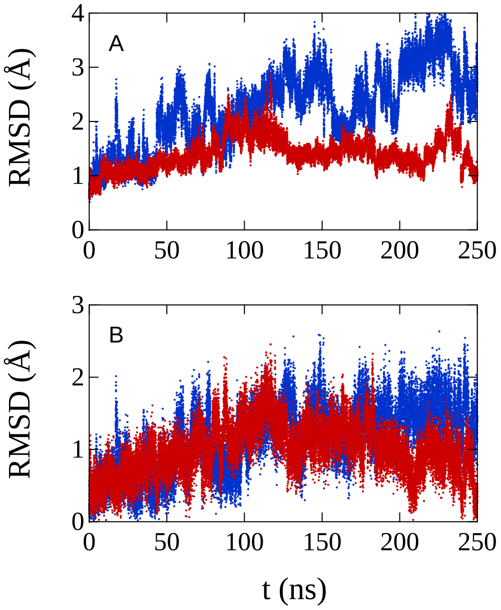

FIG. S2. RMSDs of the alpha carbons (A) and of the Fe atom (B) along the simulation trajectory. The results for the Ox (blue) and Red (red) oxidation states of the heme are shown.

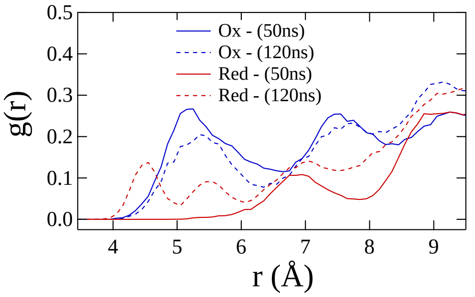

FIG. S3. The radial distribution function for the distance between the heme iron and the water's oxygen averaged over 1 ns of the simulation trajectory taken at 50 ns and 120 ns.

pocket on the time scale of the simulation when the protein is in the Ox state. This difference in wetting of the heme pocket, however, does not translate into any significant differences in the electron transfer reorganization energies in two redox states.

Induction reorganization. The standard Marcus theory was formulated for the electrostatic component of the solute-bath interaction. The equality of the two reorganization energies, $\lambda^{\mathrm{St}} = \lambda$, can be justified for this type of the interaction potential by the linear response approximation[7]. Coulomb interaction does not exhaust, however, all possible types of interactions. There are a number of nonpolar forces including dispersion interactions (often modeled by the Lennard-Jones potential)

Table S1: Excitation energies for various sizes of the QC in the Red state. QC_s and QC_m are the small and medium QCs. QC denotes the quantum center adopted for QM/MD calculations. All values are obtained by the ZINDO/S method (eV).

System	ΔE_{0-1}	ΔE_{0-2}	ΔE_{0-3}	ΔE_{0-4}	ΔE_{0-5}
QC_s	−0.23	−0.11	0.79	1.19	1.79
QC_m	0.83	0.92	1.07	1.77	1.79
QC	0.84	0.89	1.01	1.76	1.78

and induction interactions. The former asymptotically decay as r^{-6} and the latter as r^{-4} for a charge in a polarizable solvent. The relation $\lambda^{\mathrm{St}} = \lambda$ in fact does not hold for these interactions and only λ is typically of some interest since λ^{St} is close to zero[8]. The reorganization energy due to induction interactions, λ_{ind} adds to λ from the Coulomb interactions. It is typically relatively small, $\sim 0.1-0.2$ eV, and is neglected in most numerical studies.

Here we estimate $\lambda^{\mathrm{St}}_{\mathrm{ind}}$ and λ_{ind} for the cytochrome c half reaction. One of the goals of this estimate is to evaluate possible errors related to the use of non-polarizable force fields in our simulations and their correction by the *a posteriori* introduction of the induced dipoles in the analysis of trajectories.[9?] Induced dipoles create screening of electrostatic interactions and reduce the reorganization energy by $\sim$ 20% of its value in non-polarizable solvents[10]. Since dielectric screening is mostly a mean-field effect, it is essentially taken into account by enhanced mean-field dipole moments typically used in force-field potentials. However, non-zero induced dipoles also create a nonzero λ_{ind}, which off-sets the decrease due to screening. The result is that $\lambda + \lambda_{\mathrm{ind}}$ can be close to its corresponding value in a non-polarizable solvent.

In order to estimate the contribution from induction interactions, we have performed calculations according to the algorithm adopted in the past[9]. Briefly, a trajectory of the induction interaction potential with each redox states is created by calculating the free energy of inducing dipoles at atoms of the protein-water bath carrying polarizabilities α_k

$$E_i^{\mathrm{ind}} = -(1/2) \sum_k \alpha_k E_i(\mathbf{r}_k)^2, \tag{S2}$$

where $E_i(\mathbf{r}_k)$ is the electric field of the active site in the redox state $i = \mathrm{Red}, \mathrm{Ox}$ at the atom with the coordinate $\mathbf{r}_k$ and polarizability α_k. Thole's parametrization[11,12] was used for atomic polarizabilities. From the difference of averages of E_i^{ind} we find the Stokes shift reorganization energy $\lambda^{\mathrm{St}}_{\mathrm{ind}} = 0.1$ eV and from the variances in each redox state the corresponding reorganization energies $\lambda^{\mathrm{Red}}_{\mathrm{ind}} = 0.1$ eV and $\lambda^{\mathrm{Ox}}_{\mathrm{ind}} = 0.2$ eV. As mentioned, these latter values are not large enough to affect our conclusions, but can off-set a potential decrease in the reorganization energies due to screening by induced dipoles.

Polarizable active site. A portion of cytochrome c was chosen as the quantum center (QC) and was treated quantum mechanically, with the rest of the sys-

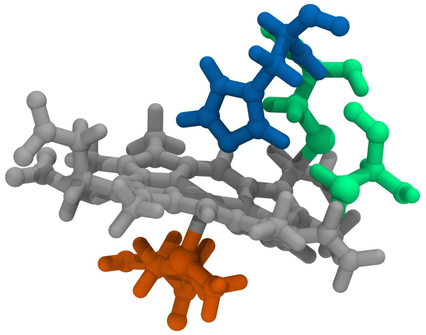

FIG. S4. Quantum center (QC): the heme group (gray) and the amino acids bonded to the heme, cysteine (green), methionine (orange), and histidine (blue).

tem treated at the classical atomistic level. Three different sizes of QC were initially chosen. The smallest QC (QC_s) only consisted of the heme. The medium size QC (QC_m) consisted of the heme, HIS, and MET ligated amino acids, and the largest QC (adopted for the analysis, Fig. S4) contained the heme, HIS, MET, and two CYS ligated amino acids. In all cases, hydrogen atoms were added to satisfy valency. Table S1 shows the unperturbed excitation energies for all QCs. It indicates that the results for the largest QC are not significantly different from QC_m. The geometry of QC was optimized by freezing all the atoms except the added hydrogens.

The Hamiltonian matrix of the QC in the electrostatic field of the surrounding classical subsystem can be written as follows[13]

$$H_{jk} = (E_j + Q\phi_{\mathrm{Fe}})\,\delta_{jk} - \boldsymbol{\mu}_{jk}\cdot\mathbf{E}_b. \tag{S3}$$

Here, Q is the total charge of the quantum center, $\boldsymbol{\mu}_{jk}$ is the transition dipole between states with energies E_j and E_k, and ϕ_{Fe} and $\mathbf{E}_b$ denoting correspondingly the electrostatic potential and the electric field of the classical subsystem at the heme iron. The multipolar terms of the order higher than the dipole are omitted here.

GAUSSSIAN'09[14] was used for all quantum calculations of the QC in vacuum (Red and Ox states) using the ZINDO/S method[15]. The charges of the Red (singlet) and Ox (doublet) QC were -2 and -1, respectively. The Hamiltonian matrix was formed by using $M = 100$ excited states. These states formed the set of parameters in Eq. (S3) and were also used to calculate the polarizability tensor of the QC according to the perturbation formula

$$\alpha_0^{\alpha\beta} = 2\sum_{j>0}\frac{\mu_{0j}^{\alpha}\mu_{j0}^{\beta}}{E_j - E_0}, \tag{S4}$$

where E_0 is the ground state energy and α, β denote the Cartesian components. Scalar polarizabilities reported in Table S2 are traces of the corresponding tensors, $\alpha = \frac{1}{3}\mathrm{Tr}\,(\boldsymbol{\alpha}_0)$.

Table S2: Scalar polarizabilities (Å^3) calculated with ZINDO/S for different numbers of excited states M, $\Delta\alpha = \alpha_{\mathrm{Ox}} - \alpha_{\mathrm{Red}}$.

M	α_{Ox}	α_{Red}	$\Delta\alpha$
100	23.3	54.1	−30.8
80	21.2	53.8	−32.6
60	18.3	52.0	−33.7
40	14.2	47.3	−33.1
20	6.5	40.6	−34.1
10	1.3	4.2	−2.9
5	3.3	3.6	−0.3

Electron transfer energy gap. The energy gap in classical MD simulations is defined as

$$X = \sum_j \Delta q_j \phi_j^{\mathrm{EW}} + X'', \tag{S5}$$

where $\Delta q_j = q_j^{\mathrm{Ox}} - q_j^{\mathrm{Red}}$, q_j^{Ox} and q_j^{Red} are the corresponding partial atomic charges in the Ox and Red states, respectively. ϕ_j^{EW} is the Ewald lattice sum electrostatic potential of the protein and water discussed below. X'' is the correction to the electrostatic energy from the interaction with the background charge of the periodic Wigner lattice[16]. The energy gap for the quantum mechanical approach is defined as the difference between the lowest eigenvalues $E_g^{\mathrm{Ox/Red}}$ of the oxidized and reduced states obtained by diagonalizing the corresponding Hamiltonian matrices in Eq. (S3)

$$X = E_g^{\mathrm{Ox}} - E_g^{\mathrm{Red}}. \tag{S6}$$

Ewald sum corrections. The treatment of long-range electrostatic interactions by NAMD software package[6] involves particle mesh Ewald sums. The simulation of redox half reactions requires, in contrast to electron transfer between the donor and acceptor, changing the charge of the system. This change in the physics of the problem demands introducing corrections to the calculations of the electrostatic energies[16–18]. Most of the corrections for the Ewald sums electrostatics discussed in the literature[18] are concerned with the equilibrium solvation free energy. The problem at hand here is different. We want to establish the corrections to the instantaneous energy gap $X(t)$ due to the use of the electrostatics produced by the lattice of replicated simulations cells, instead of an infinite polarized thermal bath.

The main point of concern in using the Ewald lattice sums for the calculation of the energy gap of a half reaction is the fact that the simulation cell is either explicitly neutralized or it is neutralized implicitly by a uniform background charge spread throughout the cell. In our present simulations, we do not use electrolyte to neutralize the simulation cell to avoid complications from the movement of the electrolyte ions. Similar setups, with no neutralizing electrolyte, were used in early work by Hummer, Pratt, and Garcia[19]. Ions are often even harder to sample adequately than the fluctuations of the multipolar polarization of the protein-water interface. The issue

is clearly demonstrated by Fig. S6, which shows that trajectories longer than 200 ns are required to sample water-protein fluctuations contributing to λ. Full sampling of ion motions would require even longer trajectories[20]. We still performed a separate simulation in the Ox state of cytochrome c to estimate the possible ion screening effect. The results from 100 ns simulations are listed in Table S3. Theses calculations include Coulomb interactions with the ions of electrolyte, in addition to protein and water included in our main production runs. Therefore, the thermal baths interacting with the active site are not identical in two sets of simulations. Nevertheless, the results are not significantly different, suggesting a weak effect of the electrolyte on the reorganization energies, in agreement with our previous simulations[20] and analytical estimates based on Debye-Hueckel electrolyte[21,22]. The absence of neutralizing electrolyte in our production runs implies that the overall simulation cell carries the charge of $Q_{\mathrm{Ox}} = 9$ and $Q_{\mathrm{Red}} = 8$ in Ox and Red states, respectively. The QC charges are correspondingly -1 and -2.

The reaction coordinate X is the difference of energies of Ox and Red state, and one can think of it as the energy difference arising from bringing a single positive charge to the Red state of the QC and distributing it over all Δq_j sites where charge density is changed in the half reaction. This extra positive charge will interact with the periodic Ewald potential ψ created by the protein-water solvent and by the lattice of replicated simulation cells. The instantaneous configuration of the bath with the set of atomic charges q_k will therefore produce the energy gap component $X' = \sum_{j \neq k} \Delta q_j \psi_{jk} q_k$, where the lattice potential is usually given as[17]

$$\psi_{jk} = \frac{\mathrm{erfc}(\kappa r_{jk})}{r_{jk}} + \frac{4\pi}{L^3} \sum_{\mathbf{k} \neq \mathbf{0}} \frac{1}{k^2} e^{i\mathbf{k}\cdot\mathbf{r}_{jk} - k^2/(4\kappa^2)} - \frac{\pi}{\kappa^2 L^3}. \tag{S7}$$

Here, $r_{jk} = |\mathbf{r}_j - \mathbf{r}_k|$, L is the side length of the cubic simulation cell, and $\mathbf{k}$ are the wavevectors of the reciprocal lattice. Correspondingly, one gets $\phi_j^{\mathrm{EW}} = \sum_k \psi_{jk} q_k$ in Eq. (S5). The lattice potential in Eq. (S7), with the last term dropped, is calculated by NAMD's "pair interactive" directive[6]; $\kappa = 0.2579$ Å^{-1} was adopted in the simulations and in the analysis of the simulaltion trajectories.

The calculation of the interaction of the fictitious positive charge transferred to the QC with the uniform background charge requires more care. The transferred charge will interact with the total charge Q_{Red} of the cell in the Red state, but will also create its own replicated images and the corresponding background charge. Those images and the corresponding background charge are not physical charges and one has to assume that they are created instantaneously as the extra charge is transferred to the cell (even though the transfer of electron is essentially instantaneous in respect to the nuclear coordinates). The corresponding contribution to the energy difference X will therefore be the free energy of charging, in contrast to the energy of interacting with the existing charge Q_{Red}. The result is

$$X'' = -\sum_j \frac{\Delta q_j^2}{2L} c' \zeta_{\mathrm{EW}} - \sum_j \frac{Q_{\mathrm{Red}} \Delta q_j}{L} c' \zeta_{\mathrm{EW}}, \tag{S8}$$

where $\zeta_{\mathrm{EW}} = 2.837297$ comes from the self-energy of a point charge in the cubic Wigner lattice[16] and $c' = 1 - \epsilon_s^{-1}$ is the correction for the "under-solvation" effects[17]. The latter correction accounts for the difference of the solvent potential in a replicated lattice compared to an infinite system. It is commonly estimated from the difference in corresponding continuum solvation energies as calculated by Hummer et al[16] and Hünenberger and McCammon[18]. Therefore, the correction c' involves the dielectric constant of the solvent ϵ_s entering the boundary value problem. When the charges Δq_j are spread over the active site immersed in a polarizable solvent with instantaneously responding electronic polarization, c' in Eq. (S8) is replaced by the Pekar factor $c_0 = \epsilon_\infty^{-1} - \epsilon_s^{-1}$, where ϵ_∞ is the electronic dielectric constant[23] (not a part of our force field).

Since $\sum_j \Delta q_j = 1$, one can simplify the above equation to

$$X'' = -\frac{c' \zeta_{\mathrm{EW}}}{2L} \sum_j \Delta q_j^2 - \frac{c' Q_{\mathrm{Red}}}{L} \zeta_{\mathrm{EW}}. \tag{S9}$$

The overall instantaneous energy gap is given by the sum of the lattice part and the interaction with the background charge, $X = X' + X''$. Since the definition of the reaction coordinate is the same in the Red and Ox sampling simulations, it is easy to see that X'' causes only a constant shift of the energy gap, which does not affect either λ^{St} or λ. We additionally note that Fig. 4A in the main text shows the distribution of the Ewald sum component X' of the reaction coordinate only, thus omitting the X'' correction. This is done to show the relative energies of interaction of the active site with the protein and water components separately, for which the corresponding corrections due to the background charge are not easy to establish. As mentioned, any changes to this procedure will only shift the corresponding distributions, without affecting the reported reorganization energies.

We also note that the effect of the system size[23] is small in our simulations. This is shown is Table S3 where we list the results of simulations of a much smaller system, with only 6626 TIP3P water molecules present in the simulation cell. Despite a smaller system size and somewhat shorter trajectories, the results are generally consistent with those obtained for a larger system.

Statistics. Two reorganization energies of electron transfer are considered here: λ^{St} and λ. The former is defined in terms of the average energy gap $\langle X \rangle$ in Red and Ox states: $\lambda^{\mathrm{St}} = \left(\langle X \rangle_{\mathrm{Red}} - \langle X \rangle_{\mathrm{Ox}}\right)/2$. The latter is given through the variance, $\lambda_i = \langle (\delta X)^2 \rangle_i / (2k_{\mathrm{B}}T)$ averaged over the configurations in equilibrium with the corresponding redox state of the protein, i = Red, Ox.

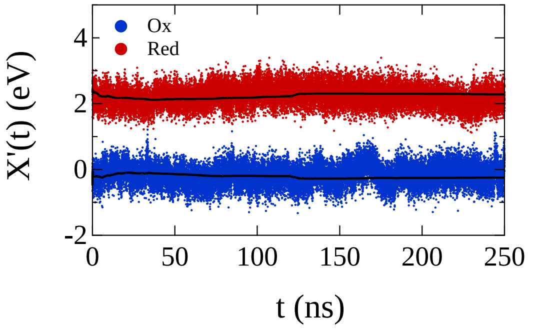

FIG. S5. Energy gap $X'(t)$ vs time excluding Ewald corrections in Ox (blue) and Red (red) states. Distributions collected from these trajectories are shown in Fig. 4A of the main text. The black curves are the accumulated average of the energy gap; the difference of the end black points gives the Stokes shift.

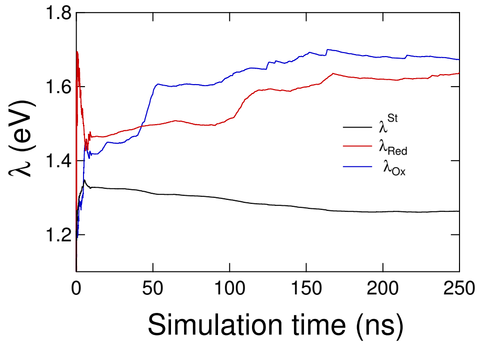

FIG. S6. The evolution of $(\langle X\rangle_{\mathrm{Red}} - \langle X\rangle_{\mathrm{Ox}})/2$ (λ^{St}) and $\beta\langle(\delta X)^2\rangle_i/2$ (λ_i, $i = \mathrm{Red, Ox}$) along classical MD trajectories obtained from simulations of cytochrome c in Ox and Red states.

Figure S5 shows the trajectory of $X(t)$ used to calculate the statistics. Table S3 reports both reorganization energies and Fig. S6 shows the evolution of λ^{St} and λ with the simulation time from classical MD simulations. The splitting of the reorganization energy λ from classical simulations into the protein and water components is also reported in Table S3. Note that λ_p (protein) and λ_w (water) do not add up to λ because the cross term λ_{pw} due to correlated protein-water fluctuations is negative and typically large in magnitude.

Figure S7 shows the free energy surfaces of the half reaction in oxidized and reduced states of cytochrome c when the polarizability change between Ox and Red states is equal to -122.9 Å^3. The corresponding free energy surfaces for $\Delta\alpha = -30.8$ Å^3 are shown in the main text.

Dynamics. The main dynamic function studied here is the time auto-correlation function of the energy gap (Stokes-shift dynamics) $C_X(t) = \langle\delta X(t)\delta X(0)\rangle$. This

Table S3: Reorganization energies (eV). The uncertainties are estimated from block averages along the simulation trajectory.

	Classical		
State	λ	λ_{p}	λ_{w}
Ox[a]	1.67 ± 0.08	2.28 ± 0.25	3.39 ± 0.27
Red[a]	1.64 ± 0.06	2.98 ± 0.24	2.46 ± 0.20
Method	λ^{St}	λ_{Ox}	λ_{Red}
Eq. S5	1.26 ± 0.04	1.67 ± 0.08	1.64 ± 0.06
Localized[b]	1.13 ± 0.04	1.57 ± 0.08	1.50 ± 0.06
Small[c]	1.04	1.24	1.49
Electrolyte[d]		1.40	
	Quantum Mechanical		
$\Delta\alpha^e$/Å^3	λ^{St}	λ_{Ox}	λ_{Red}
0.0	1.13 ± 0.04	1.57 ± 0.08	1.50 ± 0.05
-7.7	1.17 ± 0.03	2.06 ± 0.10	1.60 ± 0.06
-30.8	1.24 ± 0.03	3.07 ± 0.13	2.32 ± 0.06
-69.2	1.32 ± 0.04	4.52 ± 0.19	3.50 ± 0.09
-122.9	1.40 ± 0.05	6.40 ± 0.25	5.16 ± 0.13

[a]Based on Eq. (S5) and $\lambda = \beta\left\langle\delta X^2\right\rangle/2$, [b]Energy gap is obtained as $X = e\phi_{\mathrm{Fe}}$, where ϕ_{Fe} is the bath electrostatic potential at the heme iron, [c]Small simulation system with 21625 atoms, 6626 TIP3P waters, and with the trajectory length of 70 ns. [d]100 ns simulations with neutralizing electrolyte added to the simulation cell. [e]Based on the scaling of the transition dipole moments.

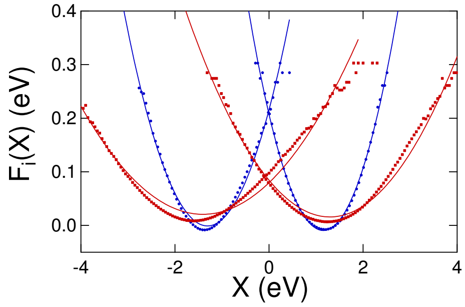

FIG. S7. Free energy surfaces of the half reaction in Ox (left) and Red (right) states of cytochrome c. The transition dipoles in the quantum calculations are scaled to produce the the polarizability change in the redox reaction: $\Delta\alpha = -122.9$ Å^3. The blue circles show the results of classical MD simulations, the red squares indicate the quantum mechanical simulations, and the solid lines are fits to parabolas.

correlation function calculated from MD trajectories in Red and Ox states of cytochrome c was fitted to five decaying exponents

$$S_2(t) = C_X(t)/C_X(0) = \sum_{n=1}^{5} A_n e^{-t/\tau_n} \qquad \text{(S10)}$$

with the fitting parameters listed in Table S4 ($\sum_{i=1}^{5} A_i = 1$). The fitted functions were then Laplace-Fourier transformed to obtain the loss function $\chi''(\omega)$ discussed in the main text. The procedure was repeated for the protein

Table S4: The fit parameters for the time correlation functions of the energy gap to the sum of 5 exponential functions (Eq. (S10), relaxation times τ_n are in ps).

Component	A_1	A_2	A_3	A_4	A_5	τ_1	τ_2	τ_3	τ_4	τ_5
Red										
Total	0.55	0.14	0.14	0.05	0.12	0.10	6.8	57	746	28380
Protein	0.14	0.05	0.13	0.25	0.44	0.06	2.3	13	106	1793
Water	0.24	0.13	0.16	0.28	0.19	0.01	4.7	78	853	5725
Ox										
Total	0.53	0.13	0.07	0.06	0.22	0.08	2.5	21	339	4685
Protein	0.15	0.10	0.16	0.14	0.45	0.01	11.4	144	813	5333
Water	0.22	0.10	0.08	0.17	0.43	0.13	2.9	23	246	4038

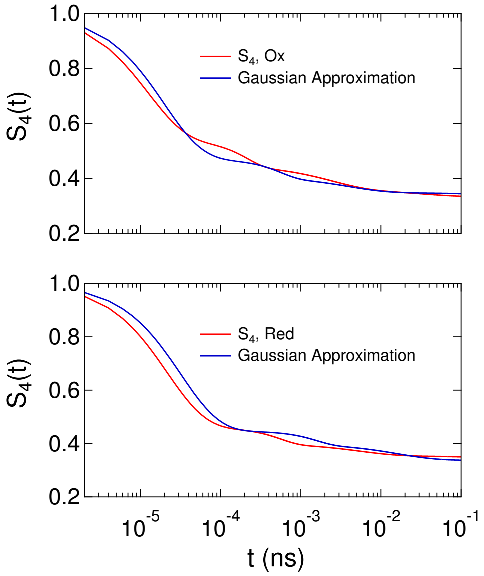

FIG. S8. $S_4(t)$ calculated from MD simulations and from Eq. (S12) (Gaussian approximation) by using the second-order correlation function $S_2(t)$ for oxidized and reduced states.

and water components of the energy gap to obtain the corresponding loss functions and the dynamics of the protein and water components of the thermal bath affecting electron transfer.

We have additionally studied the higher-order time correlation function in order to test whether the dynamics of the energy gap are Gaussian[24,25]. The normalized fourth-order time correlation functions $S_4(t)$ is defined as follows

$$S_4(t) = \langle (\delta X)^4 \rangle^{-1} \langle \delta X(t)^2 \delta X(0)^2 \rangle. \tag{S11}$$

If the dynamics are Gaussian, the fourth-order time correlation function does not carry any new dynamic information and can be determined in terms of the normalized Stokes-shift correlation function $S_2(t)$ in Eq. (S10)

$$S_4(t) = \frac{1}{3} + \frac{2}{3} S_2(t)^2. \tag{S12}$$

This relation was tested by MD simulations. Figure S8 compares $S_4(t)$ directly calculated from MD trajectories for $X(t)$ to Eq. (S12). We find a good agreement between the two results in both redox states, testifying to the accuracy of the dynamic Gaussian approximation.

References

[1] Feller, S. E., Mackerell Jr, A. D. & Mackerell, A. D. J. An Improved Empirical Potential Energy Function for Molecular Simulations of Phospholipids . *J. Phys. Chem. B* **104**, 7510–7515 (2000).

[2] Leu, B. M. *et al.* Resilience of the iron environment in heme proteins. *Biophys. J.* **95**, 5874–89 (2008).

[3] Kaszuba, K. *et al.* Parameterization of the prosthetic redox centers of the bacterial cytochrome bc 1 complex for atomistic molecular dynamics simulations. *Theor. Chem. Acc.* **132**, 1–13 (2013).

[4] Kleingardner, J. G. & Bren, K. L. Biological Significance and Applications of Heme c Proteins and Peptides. *Acc. Chem. Res.* **48**, 1845–1852 (2015).

[5] Marti, M. A., Capece, L., Crespo, A., Doctorovich, F. & Estrin, D. A. Nitric oxide interaction with cytochrome c' and its relevance to guanylate cyclase. Why does the iron histidine bond break? *J. Am. Chem. Soc.* **127**, 7721–7728 (2005).

[6] Phillips, J. C. *et al.* Scalable molecular dynamics with NAMD. *J. Comp. Chem.* **26**, 1781–802 (2005).

[7] Matyushov, D. V. Energetics of electron transfer reactions in soft condensed media. *Acc. Chem. Res.* **40**, 294–301 (2007).

[8] Matyushov, D. V. & Schmid, R. Optical and radiationless intramolecular electron transitions in nonpolar fluids: Relative effects of induction and dispersion interactions. *J. Chem. Phys.* **103**, 2034–2049 (1995).

[9] LeBard, D. N., Kapko, V. & Matyushov, D. V. Energetics and kinetics of primary charge separation in bacterial photosynthesis. *J. Phys. Chem. B* **112**, 10322–10342 (2008).

[10] Gupta, S. & Matyushov, D. V. Solvent and solute polarizability effects on the reorganization energy of electron transfer. *J. Phys. Chem. A* **108**, 2087 (2004).

[11] Thole, B. T. Molecular polarizabilities calculated with a modified dipole interaction. *Chem. Phys.* **59**, 341 (1981).

[12] van Duijnen, P. & Swart, M. Molecular and atomic polarizabilities: Thole's model revisited. *J. Phys. Chem. A* **102**, 2399–2407 (1998).

[13] Bortolotti, C. A. *et al.* The Reversible Opening of Water Channels in Cytochrome c Modulates the Heme Iron Reduction Potential. *J. Am. Chem. Soc.* **134**, 13670–13678 (2012).

[14] Frisch, M. J. *et al.* Gaussian 09 Revision E.01 (2009).

[15] Zerner, M. C., Loew, G. H., Kirchner, R. F. & Mueller-Westerhoff, U. T. An intermediate neglect of differential overlap technique for spectroscopy of transition-metal complexes. Ferrocene. *J. Am. Chem. Soc.* **102**, 589–599 (1980).

[16] Hummer, G., Pratt, L. R. & García, A. E. Ion Sizes and Finite-Size Corrections for Ionic-Solvation Free Energies. *J. Chem. Phys.* **107**, 9275–9277 (1997).

[17] Figueirido, F., Del Buono, G. S. & Levy, R. M. On finite-size effects in computer simulations using the Ewald potential. *J. Chem. Phys.* **103**, 6133–6142 (1995).

[18] Hünenberger, P. H. & McCammon, J. A. Ewald artifacts in computer simulations of ionic solvation and ion–ion interaction: A continuum electrostatics study. *J. Chem. Phys.* **110**, 1856 (1999).

[19] Hummer, G., Pratt, L. R. & Garcia, A. E. Free energy of ionic hydration. *J. Phys. Chem.* **100**, 1206–1215 (1996).

[20] Martin, D. R. & Matyushov, D. V. Non-Gaussian statistics and nanosecond dynamics of electrostatic fluctuations affecting optical transitions in a green fluorescent protein. *J. Phys. Chem. B* **116**, 10294–10300 (2012).

[21] German, E. D. & Kuznetsov, A. M. Influence of ionic strength on charge transfer processes in polar media. *Electrokhimiya* **28**, 294 (1992).

[22] Zhu, J., Ma, R., Lu, Y. & Stell, G. Dynamic salt effect on intramolecular charge-transfer reactions. *J. Chem. Phys.* **123**, 224505 (2005).

[23] Ayala, R. & Sprik, M. A Classical Point Charge Model Study of System Size Dependence of Oxidation and Reorganization Free Energies in Aqueous Solution . *J. Phys. Chem. B* **112**, 257–269 (2008).

[24] Dinpajooh, M. & Matyushov, D. V. Non-Gaussian lineshapes and dynamics of time-resolved linear and nonlinear (correlation) spectra. *J. Phys. Chem. B* **118**, 7925–7936 (2014).

[25] Roy, S., Pshenichnikov, M. S. & Jansen, T. L. C. Analysis of 2D CS spectra for systems with non-gaussian dynamics. *J. Phys. Chem. B* **115**, 5431–40 (2011).
